# 

**Published:** 1914-12-05

**Authors:** 


### LEICESTER ROYAL INFIRMARY.

### Personalia.

The picture of Viscount Knutsford now in the board room has been presented to him by the House Committee, and by him to the London Hospital, as a mark of their appreciation of his splendid services on behalf of the hospital during the nineteen years he has been its chairman. It was painted by Mr. Oswald Birley, who is now fighting as a private at the Front.

Sir Frederick Eve, senior surgeon to the London Hospital, has sent in his resignation as from the beginning of December, when he completed a period of thirty years' service on the surgical staff. The committee, in receiving Sir Frederic Eve's resignation, has unanimously elected him consulting surgeon to the London Hospital.

### Gifts and Bequests.

The Chelsea Hospital for Women has received from the executors of the will of the late Mr. Thomas Stephen Whitaker the sum of £2,000 towards the rebuilding of the new hospital.

Recent legacies to the Leicester Royal Infirmary include bequests of £500 to the Children's Hospital, under the will of the late Mr. Alfred Green; £50 to the infirmary, under the will of Miss Ann Wilford Preston, of Long Clawson; and £5 under the will of the late Mr. John Staples.

THE CONVALESCENT HOMES' ASSOCIATION'S GRANTS.

The following grants have been made by the Convalescent Homes Association to affiliated Homes, having some special need, from the "Louisa Sophia Lady Goldsmid" Fund and the surplus funds of the Association: Baldwin Brown Homes, Herne Bay, £10; "Clara, Baroness de Hirsch" Home, Hampstead, £20; Boys' Surgical Home, Banstead, £20; Brentwood Children's Convalescent Home, £10; "Catherine" House Home for Invalid Gentlewomen, Hastings, £25; "Catherine Gladstone" Free Convalescent Home, Mitcham, £15; Children's Home, Beaconsfield, £10; Children's Home, Glynde, £10; Children's Home Hospital, Barnet, £20; Hahnemann Home, Bournemouth, £15; Convalescent Home for Poor Children, St. Leonards, £10; "Winifred" House Invalid Children's Hospital Home, Tollington Park, £10; St. Mary's Home, Birchington, £20; St. Oswald's Home, Brighton, £15; "Sunshine" Homes, Hassocks, £20; Victoria Home for Invalid Children, Margate, £20. With a previous distribution of £146 10s. in grants to nine Homes in 1912, the total sum allocated to affiliated institutions from the funds of the Association available for the purpose now amounts to £396 10s. Mr. Alex. Hayes is the Secretary of the Association, whose offices are at 14 Victoria Street, S.W.

### Buildings Contemplated.

Alton.—The Guardians have been authorised to purchase a site adjoining the workhouse for a new infirmary at a cost of £1,000.

Cleadon.—The South Shields Town Council have resolved to purchase twelve acres adjoining the site of the infectious hospital at Cleadon for proposed extensions.

Heston.—The Middlesex County Council have decided to purchase Rectory Farm and part of Vicarage Farm for a new county asylum, and to use a portion of the site for an institution for mental defectives under the Mental Deficiency Act.

Hexham.—It is proposed to build a new hospital at Lough Lane.

Milford.—The Surrey County Council have received the sanction of the Local Government Board to the purchase of the Sattenham Estate, at a cost of £8,000, for a site for a sanatorium and hospital.

Newcastle.—The Staffordshire, Wolverhampton, and Dudley Joint Committee for Tuberculosis have approved the purchase of land in Friar's Wood Road as a site for a hospital and a dispensary.

Norton.—The Stockton Guardians have received the sanction of the Local Government Board to borrow £1,750 for the purchase of the site in Junction Lane on which the new workhouse infirmary will be built.

Ragwell.—The East Riding County Council have decided to enter into a provisional agreement for the purchase of property at Ragwell for the provision of a sanatorium. The estimated cost is £13,075.

Sheffield.—The Corporation Finance Committee have approved the expenditure of £20,000, exclusive of £2,750 for the site, for building a tuberculosis sanatorium for women in Rivelin Valley Road. Application will be made to the Local Government Board for sanction to borrow the amount.

Spittlesea.—The Luton Town Council have approved a scheme for the extension of the Spittlesea Hospital at a cost of £5,320.

Willian.—The Herts County Council have approved plans for a sanatorium and hospital on a site at Willian at an estimated cost of £27,662 12s. 3d.

### The Book Market.

LIST OF NEW BOOKS RECEIVED FROM THE FOLLOWING PUBLISHERS:—

Edward Arnold: "Medical Nursing." By A. S. Woodwark, M.D. Price 4s. 6d. net.

Constable and Co., Ltd.: "The Acute Abdomen." Second Edition. By W. H. Battle, Senior Surgeon to St. Thomas's Hospital. Price 10s. 6d. net.

T. Werner Laurie, Ltd.: "The Soldiers' English-French Conversation Book." Price 7d. net. "Nietzsche." By J. M. Kennedy. Price 1s. net.

The Clinical Research Association, Ltd.: "The Practitioners' Guide to Clinical Research."

Rates of Subscription : Twelve months, 6/6 ; Six months, 4/- ; Three months, 2/-, post free to any address in the United Kingdom. Abroad, Twelve months, 8/8 ; Six months, 5/- ; Three months, 2/6, post free.—Address The Subscription Dept., "The Hospital," The Hospital Building, 28/29 Southampton Street, Strand, London, W.C.

